# Screening of the ‘Stasis Box’ identifies two kinase inhibitors under pharmaceutical development with activity against *Haemonchus contortus*

**DOI:** 10.1186/s13071-017-2246-x

**Published:** 2017-07-05

**Authors:** Yaqing Jiao, Sarah Preston, Anson V. Koehler, Andreas J. Stroehlein, Bill C. H. Chang, Kaylene J. Simpson, Karla J. Cowley, Michael J. Palmer, Benoît Laleu, Timothy N. C. Wells, Abdul Jabbar, Robin B. Gasser

**Affiliations:** 1 Faculty of Veterinary and Agricultural Sciences, Melbourne Veterinary School, The University of Melbourne, Parkville, VIC 3010 Australia; 2 Victorian Centre for Functional Genomics, Peter MacCallum Cancer Centre, Parkville, VIC Australia; 3 The Sir Peter MacCallum Department of Oncology, University of Melbourne, Parkville, VIC Australia; 4 Medicines for Malaria Venture (MMV), Route de Pré-Bois 20, CH-1215 Geneva, Switzerland

**Keywords:** Medicines for Malaria Venture (MMV), Stasis Box, Repurposing, Anthelmintic, Nematodes, *Haemonchus contortus*, Whole-organism screen

## Abstract

**Background:**

In partnership with the Medicines for Malaria Venture (MMV), we screened a collection (‘Stasis Box’) of 400 compounds (which have been in clinical development but have not been approved for illnesses other than neglected infectious diseases) for inhibitory activity against *Haemonchus contortus*, in order to attempt to repurpose some of the compounds to parasitic nematodes.

**Methods:**

We assessed the inhibition of compounds on the motility and/or development of exsheathed third-stage (xL3s) and fourth-stage (L4) larvae of *H. contortus* using a whole-organism screening assay.

**Results:**

In the primary screen, we identified compound MMV690767 (also known as SNS-032) that inhibited xL3 motility by ~70% at a concentration of 20 μM after 72 h as well as compound MMV079840 (also known as AG-1295), which induced a coiled xL3 phenotype, with ~50% inhibition on xL3 motility. Subsequently, we showed that SNS-032 (IC_50_ = 12.4 μM) and AG-1295 (IC_50_ = 9.92 ± 1.86 μM) had a similar potency to inhibit xL3 motility. Although neither SNS-032 nor AG-1295 had a detectable inhibitory activity on L4 motility, both compounds inhibited L4 development (IC_50_ values = 41.24 μM and 7.75 ± 0.94 μM for SNS-032 and AG-1295, respectively). The assessment of the two compounds for toxic effects on normal human breast epithelial (MCF10A) cells revealed that AG-1295 had limited cytotoxicity (IC_50_ > 100 μM), whereas SNS-032 was quite toxic to the epithelial cells (IC_50_ = 1.27 μM).

**Conclusions:**

Although the two kinase inhibitors, SNS-032 and AG-1295, had moderate inhibitory activity on the motility or development of xL3s or L4s of *H. contortus* in vitro, further work needs to be undertaken to chemically alter these entities to achieve the potency and selectivity required for them to become nematocidal or nematostatic candidates.

**Electronic supplementary material:**

The online version of this article (doi:10.1186/s13071-017-2246-x) contains supplementary material, which is available to authorized users.

## Background

Parasites of animals cause diseases of major socioeconomic importance globally [1, 2]. For example, gastrointestinal nematodes of livestock cause subclinical infections and diseases that lead to reductions in meat, milk and fibre production [3, 4], with at least AUD $500 million losses per annum in Australia alone [5]. Thus, parasitic nematode infections are a substantial burden to animal health and livestock production. Currently, anthelmintic treatment remains the mainstay of control for parasitic nematodes [6, 7]. The occurrence of anthelmintic resistance, together with the limited number of anthelmintics being commercialised, indicates an urgency to discover new and effective anthelmintic compounds [6, 8, 9].

Product development partnerships (PDPs) are playing a significant role in the development of new medicines for neglected diseases [10, 11]. In the present context, a PDP usually involves a collaboration between a non-profit organisation, such as the Medicines for Malaria Venture (MMV), Drugs for Neglected Diseases Initiative (DNDi) and Global Alliance for TB Drug Development (TB Alliance), industry and/or academic partners to collectively combat infectious/parasitic diseases [10, 12]. The significant role of the PDP model is demonstrated through the delivery of commercial products, such as paromomycin against leishmaniasis, developed by the Institute of One World Health [13], artemether-lumefantrine (Coartem dispersible) - a child-friendly treatment against malaria developed in partnership by Novartis and MMV [14], and a new vaccine called MenAfriVac against the bacterial meningitis by the Meningitis Vaccine Project [15, 16].

Recently, in collaboration with MMV, we screened compounds in the ‘Pathogen Box’ with known activities against one or more pathogens that cause neglected diseases (including tuberculosis, malaria, sleeping sickness, leishmaniasis, schistosomiasis, hookworm disease, toxoplasmosis and cryptosporidiosis) against *Haemonchus contortus* [17], an economically important parasitic nematode of ruminants that represents a large order of nematodes, the Strongylida [18]. We identified tolfenpyrad, an approved pesticide with known activity against some kinetoplastid protists [19], which has anthelmintic activity against *H. contortus* [17]. Within this collaborative framework, we were able to source another library, called the ‘Stasis Box’, from MMV, which contains 400 compounds that have been in clinical development but have not been approved for illnesses other than neglected infectious diseases. The ‘Stasis Box’ contains compounds that have been developed against disorders such as atherosclerosis, restenosis, pulmonary fibrosis, selected cancers, urinary incontinence or depression (Mike Palmer, personal communication). Here, we screened all of these compounds against *H. contortus* using an established whole-organism motility assay [20], with the aim of repurposing some of them as nematocides.

## Methods

### Procurement of *H. contortus*

In accordance with institutional animal ethics guidelines (permit no. 1413429; The University of Melbourne), *H. contortus* (Haecon-5 strain) was maintained in experimental sheep as described previously [21]. To produce exsheathed third-stage larvae (xL3s), infective L3s were exposed to 0.15% (*v*/v) of sodium hypochlorite (NaClO) for 20 min at 37 °C [21], washed five times in sterile saline (0.9%) and cultured in Luria Bertani medium (LB) supplemented with final concentrations of 2 μg/ml of amphotericin, 100 μg/ml of streptomycin and 100 IU/ml of penicillin (antibiotic-antimycotic, cat. no. 15240–062; Life Technologies, Carsbad, USA) (LB*). To produce fourth-stage larvae (L4s), xL3s were incubated in a water-jacketed CO_2_ incubator (model no. 2406 Shel Lab, Cornelius, USA) for 7 days at 38 °C and 10% *v*/v CO_2_ or until ≥ 70% of L3s had developed to the L4 stage.

### Compound library and screening

From MMV, we obtained the ‘Stasis Box’, which contains 400 compounds that have been in drug development, as explained earlier. These compounds were individually solubilised in 10 μl of dimethyl sulfoxide (DMSO) to achieve a stock concentration of 10 mM, and then diluted and tested for activity against *H. contortus*. The compounds were tested using a previously described method [20]. In brief, using 96-well flat bottom plates, individual compounds (40 μM) in LB* (50 μl) were added to individual wells in triplicate, with LB* + 0.5% DMSO as a negative control and a commercial anthelmintic, monepantel (Zolvix, Novartis Animal Health, Basel, Switzerland) as a positive control. Subsequently, 300 xL3s in 50 μl LB* were added to individual wells. The plates were then incubated in a 38 °C water-jacketed CO_2_ incubator for 72 h. Then, a video recording (5 s) was made of each well using a grey-scale camera (Rolera bolt, Q imaging Scientific Coms, Surrey, Canada) and a motorised X-Y axis stage (BioPoint 2; Ludl Electronics Products, Hawthorne, USA). The motility of worms in each well was calculated in a pixel-based algorithm, called motility index (Mi), based on the light intensity changes caused by the worm movement [21]. For each compound, Mi values were normalized against the positive and negative controls using the program GraphPad Prism (v.6 GraphPad Software, USA). A compound was identified as a “hit” if it reduced worm motility by ≥70% or induced a phenotype that differed from wild-type xL3 (i.e. LB* + 0.5% DMSO control). For each compound, each data point represented the mean of a triplicate (± standard error of the mean, SEM).

### Dose-response assay

Active compounds (99.9% purity; purchased from Selleck Chemicals, Boston, or Cayman Chemicals, Ann Arbor, USA), were serially (two-fold) diluted from 100 μM to 0. 76 nM in triplicate in a 96-well flat bottom plate and ~300 xL3s (in 50 μl LB*) added to each well. The plates were then incubated in a 38 °C water-jacketed CO_2_ incubator and the Mi values of worms measured [20]. In addition, following the measurement of xL3 motility, L4 development rates were measured, with 30 worms from each well being examined at 20-times magnification following the addition of 50 μl of 1% iodine to each well after seven days of incubation prior to light microscopic examination at 100-times magnification. Half the maximum inhibitory concentration (IC_50_) on xL3 motility, L4 motility and L4 development were determined from the dose-response curves using a variable slope four-parameter equation in GraphPad Prism by constraining the top value to 100% and using a least squares (ordinary) fit model. For each curve, each data point represented the mean of two to five experiments repeated in triplicate on different days (± standard error of the mean, SEM).

### Assessing cytotoxicity and selectivity

Compounds with activity on *H. contortus* were tested for cell toxicity properties on a non-cancerous (‘normal’) mammary epithelial cell line (MCF10A) [22]. In brief, MCF10A cells were dispensed into wells of flat bottom 384-well, black walled plates (Corning, New York, USA) at 700 cells per well (40 μl) using a liquid handling dispenser (BioTek, Vermont, USA). Cells were cultured in DMEM-F12 containing 100 ng/ml cholera toxin (Sigma-Aldrich, St Louis, USA), 20 ng/ml human epidermal growth factor (EGF, Life Technologies, Carsbad, USA), 10 μg/ml insulin (human; Novo Nordisk Pharmaceuticals Pty Ltd., Bagsværd, Denmark), 5% horse serum (Life Technologies, Australia) and 0.5 μg/ml hydrocortisone (Sigma-Aldrich, St Louis, USA). Following incubation (24 h at 37 °C and 5% CO_2_), the growth medium was aspirated and the cells were treated with test compounds starting at 100 μM as well as positive- (monepantel or moxidectin) and negative- (medium ± 1% DMSO) controls. Compounds were titrated to generate a 5-point dose-response curve (in quadruplicate) employing an automated liquid handling robot (SciClone ALH3000 Lab Automation Liquid Handler, Caliper Life Sciences, Hopkinton, USA) and incubated for a further 48 h. For each compound concentration, matched DMSO concentrations were also tested separately to account for DMSO-induced cytotoxicity. To measure cell proliferation, cells were fixed and stained with 4′,6-diamidino-2-phenylindole (DAPI; 1:1000) and individual wells imaged at 10-times magnification, covering 16 fields (~90% of well) using a high content imager (Cellomics Cell Insight Personal Cell Imager, ThermoFisher Scientific, Bartlesville, USA) at a fixed exposure time of 0.12 s. Viable cells were counted using the Target Activation BioApplication within the Cellomics Scan software and normalized to the cell density in wells without compound. Toxicity due to DMSO was removed from the normalized cell density counts, and IC_50_ calculated from the variable slope four-parameter equation in GraphPad Prism. Experiments were repeated twice on two different days. The selectivity indices of active compounds were calculated as follows: selectivity index = human epithelial (MCF10A) cells IC_50_/*H. contortus* IC_50_ (for xL3 motility, L4 motility and L4 development).

## Results and discussion

**Fig. 1:**
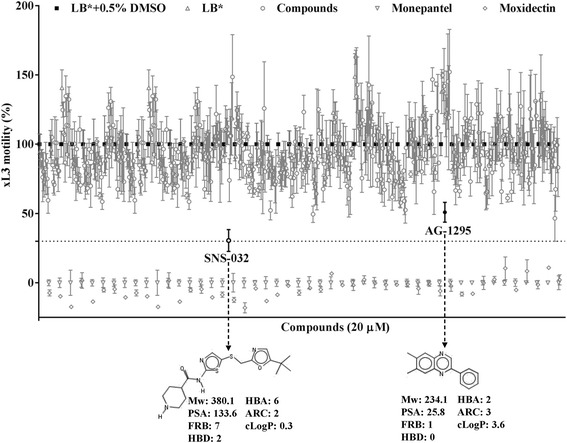
Primary screen of 400 individual compounds from the ‘Stasis Box’ from the Medicines for Malaria Venture (MMV) at a concentration of 20 μM identified compound SNS-032 (MMV690767) to inhibit the motility of exsheathed third-stage larvae (xL3) of *Haemonchus contortus* (at 72 h) by ≥ 70% compared with negative (LB* + 0.5% dimethyl sulfoxide; DMSO) and positive controls (monepantel). Another compound, AG-1295 (MMV079840), was found to inhibit xL3 motility by ~50%, displaying a “coiled” phenotype based on visual inspection of video recordings (see Additional file 1). Other compounds with apparent inhibition of ≥ 50% did not exhibit a characteristic phenotype by visual inspection. Each data point represents the mean of a triplicate (± standard error of the mean, SEM). Chemical structures and physiochemical properties of ‘hit’ compounds SNS-032 and AG-1295 are indicated. *Abbreviations*: Mw, molecular weight; PSA, polar surface area; FRB, freely rotating bonds; HBD, hydrogen bond donor; HBA, hydrogen bond acceptor; ARC, aromatic ring count; cLogP, calculated partition coefficient

In the primary screen of the 400 compounds from the ‘Stasis Box’ (Fig. 1), one compound, MMV690767 (also known as SNS-032), inhibited xL3 motility by ~70% and another compound, MMV079840 (also known as tyrphostin AG-1295 or NSC 380341), induced a coiled larval phenotype and inhibited motility by 50% (Additional file 1). No other compound inhibited motility by ≥ 70% or induced a non-wildtype phenotype. The chemical structures and predicted physicochemical properties of SNS-032 and AG-1295 are given in Fig. 1.

**Fig. 2:**
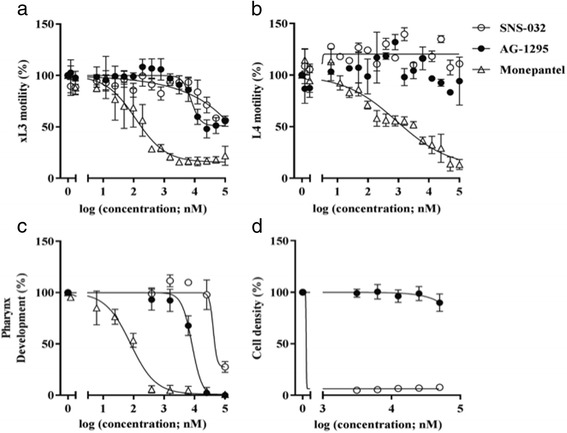
Dose-response curves for compounds SNS-032 (MMV690767) and AG-1295 (MMV079840) on larval stages of *Haemonchus contortus* in vitro with reference to the positive-control compound monepantel. Inhibition of motility of (**a**) third-stage (xL3) and (**b**) fourth-stage (L4) larvae at 72 h and of development of fourth-stage larvae (L4s) at 7 days (**c**) of exposure to each of the compound. Assessment of the toxicity of compounds SNS-032 (MMV690767) and AG-1295 (MMV079840) on breast epithelial (MCF10A) cells after 48 h of exposure to each compound in vitro (**d**). Each data point represents the mean of two to five experiments repeated in triplicate on different days (± standard error of the mean, SEM)

Compound SNS-032, a N-(5-{[(5-tert-butyl-1,3-oxazol-2-yl)methyl]sulfanyl}-1,3-thiazol-2-yl)-4-piperidinecarboxamide was developed as a cyclin dependent kinase (CDK)-2, −7 and −9 inhibitor for the treatment of B-cell lymphoma by the company Sunesis Pharmaceuticals (South San Francisco, USA) and entered phase I clinical trials. Compound AG-1295, a 6,7-dimethyl-2-phenylquinoxaline, is a protein tyrosine kinase (PTK) inhibitor targeting the platelet-derived growth factor (PDGF) receptor kinase. Given their activity against *H. contortus*, compounds SNS-032 and AG-1295 were selected for further evaluation. Dose-response curves on xL3 motility showed that SNS-032 (IC_50_ = 12.4 μM at 72 h) and AG-1295 (IC_50_ = 9.92 ± 1.86 μM at 72 h) had a similar potency at inhibiting xL3 motility, without a statistically significant difference (see Table 1; Fig. 2a). Although neither SNS-032 nor AG-1295 had any detectable inhibitory activity on L4 motility (Table 1; Fig. 2b), both compounds had considerable potency at inhibiting L4 development, with SNS-032 being less potent at inhibiting larval development than AG-1295 (IC_50_ = 41.24 μM *versus* 7.75 ± 0.94 μM for SNS-032 and AG-1295, respectively) (Table 1; Fig. 2c). Comparative IC_50_ values for monepantel (control compound) against xL3 motility, L4 motility and L4 development were 0.16 ± 0.08 μM (72 h), 0.37 ± 0.32 μM (72 h) and 0.075 ± 0.04 μM (7 days), respectively (Table 1). The testing of the two compounds for toxic effects on breast epithelial (MCF10A) cells revealed AG-1295 to have limited cytotoxicity (IC_50_ > 100 μM), whereas SNS-032 was quite toxic to these epithelial cells (IC_50_ = 1.27 μM) and not selective for the parasite (Table 2; Fig. 2d). The limited inhibitory effect of AG-1295 on the proliferation of MCF10A cells seems to associate with limited expression/transcriptional of genes encoding PDGFR-β in this non-tumorigenic cell line [23].

**Table 1:** Half of the maximum inhibition concentration (IC_50_) values for compounds SNS-032 (MMV690767) and AG-1295 (MMV079840) on the motility of exsheathed third-stage larvae (xL3) and fourth-stage-larvae (L4) of *Haemonchus contortus* (after 72 h of exposure the compound) and on the development of L4 (7 days of exposure)

Compound	IC_50_ (μM)		
	xL3 motility (72 h)	L4 motility (72 h)	L4 development (7 days)
SNS-032	12.36^a^	na	41.24^a^
AG-1295	9.92 ± 1.86	na	7.75 ± 0.94
Monepantel	0.16 ± 0.08	0.37 ± 0.32	0.075 ± 0.04

*Abbreviation*: *na* no activity

^a^Half maximum inhibitory concentration could not be accurately calculated by the log (agonist) *versus* response-variable slope (four parameter) equation, a IC_50_ value was estimated

**Table 2:** Compounds SNS-032 (MMV690767) and AG-1295 (MMV079840) were tested for toxic effects on breast epithelial (MCF10A) cells. Selectivity indices of these compounds on the motility of exsheathed third-stage larvae (xL3) and fourth-stage larvae (L4) of *Haemonchus contortus* (after 72 h of exposure to the compound) and the development of L4s (over 7 days of exposure) were calculated using a recognized formula [38]

Compound	IC_50_ (μM) for MCF10A cells	Selectivity indices for *H. contortus*		
		xL3 motility (72 h)	L4 motility (72 h)	L4 development (7 days)
SNS-032	1.3	nd	nd	0.04
AG-1295	> 100	10.1^a^	nd	10.9^a^
Monepantel	27.8	173.6	75.1	370.3

*Abbreviation*: *nd* not determined

^a^Selectivity indices were calculated based on the highest value in the IC_50_ range

**Fig. 3:**
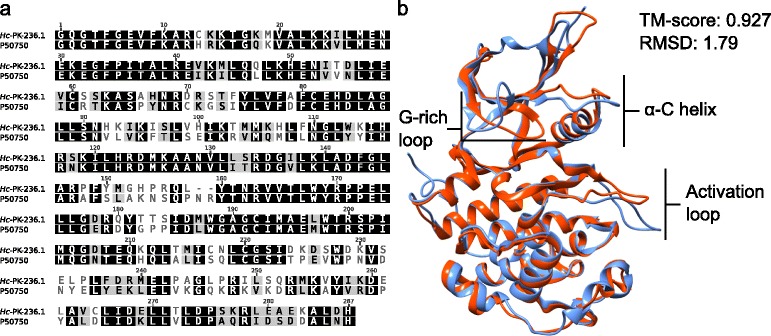
Multiple-sequence alignment showing levels of similarity in the kinase catalytic domains (Pfam identifier: PF00069) of CDK9 homologs between *Haemonchus contortus* (*Hc*-PK-236.1) and human (UniProt accession no.: P50750). **a** The pairwise sequence alignment was constructed using the program MUSCLE [39]. **b** Three-dimensional model of CDK9 homolog of *H. contortus* (*Hc*-PK-236.1; *orange*) superimposed on to the crystal structure of human CDK9 (protein data bank (PDB) identifier: 4or5A; *blue*). The TM-score and root-mean-square deviation (RMSD) values indicate a high-confidence prediction. Conformational differences predicted in G-rich loop, activation loop and α-C helix between the two structures are indicated. The three-dimensional structure for *Hc*-PK-236.1 was predicted using the program I-TASSER [40] using default parameters

SNS-032 is an anti-cancer protein kinase inhibitor that acts as an apoptosis stimulator, cell cycle inhibitor and radio-sensitizer [24–26]. Based on the current literature [27–29], SNS-032 selectively targets human cyclin-dependent kinases (CDKs), including CDK2, CDK7 and CDK9, suggesting that one or more CDKs of *H. contortus* are target(s) for this compound. This statement is also supported by a recent prediction and prioritization that CDK7 and CDK9 homologs (designated *Hc-*PK-002.1 and *Hc-*PK-236.1, respectively) of *H. contortus* are amongst the top-ten kinase drug targets for this nematode [30]. The relatively close sequence (79.4%) and structural homologies (root-mean-square deviation, RMSD: 1.79 Å) in the catalytic domain between human CDK9 and its *H. contortus* homolog (Fig. 3) appear to reflect the toxicity of SNS-032 to cells of both organisms and its limited selectivity. In addition, the subtle conformational difference predicted within the ATP-binding site of *Hc*-PK-236.1 and human CDK9 (Fig. 3) might explain a reduced potency of SNS-032 in *H. contortus* with respect to human cells (Tables 1 and 2). This information indicates that SNS-032 would need to undergo medicinal chemistry optimization to achieve high potency and selectivity for *H. contortus* and/or related nematodes before it could be considered as an anthelmintic candidate.

On the other hand, AG-1295 had more selective and better anthelmintic activity against *H. contortus* than SNS-032 (Tables 1 and 2), achieving IC_50_ values of 9.92 ± 1.86 μM (xL3 motility) and of 7.75 ± 0.94 μM (L4 development). This selectivity likely relates to limited expression of the target in (normal) MCF10A cells compared with a distinct and moderate activity in developing larvae of *H. contortus* proposed to be associated with relatively high levels of PTK expression [23, 30]. AG-1295 is a quinoxaline compound that acts as a platelet-derived growth factor (PDGF) receptor kinase inhibitor [31–33] which has been shown to attenuate porcine and human smooth muscle cell growth in vitro and to possess considerable anti-restenosis effects in pigs [34, 35]; this chemical has also been reported to significantly inhibit aortic allograft vasculopathy in rats [36] and attenuates the proliferation of rat hepatic stellate cells [37]. Current evidence shows that AG-1295 selectively inhibits PDGF receptor tyrosine kinase activity apparently without interacting with other protein kinases [31, 34, 35], and inhibits PDGF-stimulated DNA synthesis with an IC_50_ value of 2.5 μM, without affecting the activity of the epidermal growth factor (EGF) receptor [31]. Interestingly, although AG-1295 inhibited xL3 motility and L4 development in *H. contortus*, there is presently no evidence of a PDGF receptor kinase in *H. contortus* [30], suggesting an alternative kinase target. Possible targets of AG-1295 in this nematode might be one or more of five related kinases, namely *Hc-*PK-144.1 within the fibroblast growth factor receptor (FGFR) tyrosine kinase family, *Hc-*PK-185.1 and *Hc-*PK-200.1 within the growth factor receptor tyrosine kinase-like family KIN16 (similar to human vascular endothelial growth factor, VEGFR), and *Hc-*PK-319.1 and *Hc-*PK-319.2 within the EGF receptor tyrosine kinase family [30]. Further work needs to be done to test these proposals using an integrated experimental-structural biology approach.

## Conclusions

Although two kinase inhibitors (SNS-032 and AG-1295) were identified and shown to have moderate inhibitory activity on the motility or development of xL3s or L4s of *H. contortus* in vitro, substantial further work would need to be undertaken to chemically modify these entities to achieve the potency and selectivity needed for them to become viable nematocidal or nematostatic drug candidates.

## Additional file

Additional file 1: Five-second video recordings of exsheathed third-stage larvae (xL3s) of *Haemonchus contortus* displaying the “coiled” phenotype induced by exposure to AG-1295 (MMV079840; 100 μM). Videos of xL3s exposed to the same concentration of SNS-032 (MMV690767), monepantel, or no-compound were also included for comparison. (PPTX 18505 kb)

## Abbreviations

IC_50_: half maximum inhibitory concentration values
L4: fourth-stage larvae
LB: Luria Bertani medium
MCF10A cells: Human breast epithelial cells
Mi: Motility index
MMV: Medicines for Malaria Venture
xL3: exsheathed third-stage larvae

## References

[1] Anderson RC. Nematode Parasites of Vertebrates: Their Development and Transmission. Second edition. CAB; 2000.

[2] Beveridge I, Emery D. Australian Animal Parasites - Inside and Out. The Australian Society for Parasitology Inc. Australia; pp. 8–19. 2014. ISBN 978-0-646-93560-7.

[3] Charlier J, van der Voort M, Kenyon F, Skuce P, Vercruysse J (2014). Review: chasing helminths and their economic impact on farmed ruminants. Trends Parasitol.

[4] Preston SJ, Sandeman M, Gonzalez J, Piedrafita D (2014). Current status for gastrointestinal nematode diagnosis in small ruminants: where are we and where are we going?. J Immunol Res.

[5] Lane J, Jubb T, Shephard R, Webb-Ware J, Fordyce G. Priority list of endemic diseases for the red meat industries. Meat & Livestock Australia Limited (MLA). 2015; ISBN: 9781741918946; pp. 182.

[6] Epe C, Kaminsky R (2013). New advancement in anthelmintic drugs in veterinary medicine. Trends Parasitol.

[7] Geary TG, Sakanari JA, Caffrey CR (2015). Anthelmintic drug discovery: into the future. J Parasitol.

[8] Kaplan RM, Vidyashankar AN (2012). An inconvenient truth: global worming and anthelmintic resistance. Vet Parasitol.

[9] Kotze AC, Prichard RK (2016). Anthelmintic resistance in *Haemonchus contortus*: history, mechanisms and diagnosis. Adv Parasitol.

[10] Moran M, Guzman J, Ropars AL, Illmer A. The role of product development partnerships in research and development for neglected diseases. Int Health. 2010;2:114–22. 10.1016/j.inhe.2010.04.002 24037470

[11] Reeder JC, Mpanju-Shumbusho W (2016). Building research and development on poverty-related diseases. Bull World Health Organ.

[12] Grace C. Product development partnerships (PDPs): lessons from PDPs established to develop new health technologies for neglected diseases, Department for International Development, UK; USAID Report to Congress: Coordinate Strategy to Accelerate Development of Vaccines for Infectious Diseases, USA; 2010.

[13] Davidson RN, den Boer M, Ritmeijer K (2009). Paromomycin. Trans R Soc Trop Med Hyg.

[14] Premji ZG (2009). Coartem: the journey to the clinic. Malar J.

[15] Butler D (2010). Vaccine offers meningitis hope. Nature.

[16] Bishai DM, Champion C, Steele ME, Thompson L (2011). 2011. Product development partnerships hit their stride: lessons from developing a meningitis vaccine for Africa. Health Aff.

[17] Preston S, Jiao Y, Jabbar A, McGee SL, Laleu B, Willis P (2016). Screening of the ‘Pathogen Box’ identifies an approved pesticide with major anthelmintic activity against the barber's pole worm. Int J Parasitol Drugs Drug Resist..

[18] Schwarz EM, Korhonen PK, Campbell BE, Young ND, Jex AR, Jabbar A (2013). The genome and developmental transcriptome of the strongylid nematode *Haemonchus contortus*. Genome Biol.

[19] Witschel M, Rottmann M, Kaiser M, Brun R (2012). Agrochemicals against malaria, sleeping sickness, leishmaniasis and Chagas disease. PLoS Negl Trop Dis.

[20] Preston S, Jabbar A, Nowell C, Joachim A, Ruttkowski B, Cardno T (2016). Practical and low cost whole-organism motility assay: a step-by-step protocol. Mol Cell Probes.

[21] Preston S, Jabbar A, Nowell C, Joachim A, Ruttkowski B, Baell J (2015). Low cost whole-organism screening of compounds for anthelmintic activity. Int J Parasitol.

[22] Kumarasingha R, Karpe AV, Preston S, Yeo TC, Lim DSL, Tu C-L, et al. Metabolic profiling and in vitro assessment of anthelmintic fractions of *Picria fel-terrae* Lour. Int J Parasitol Drugs Drug Resist. 2016;6:171–8. 10.1016/j.ijpddr.2016.08.002 PMC5030326 27639945

[23] Kadivar A, Kamalidehghan B, Akbari Javar H, Karimi B, Sedghi R, Noordin MI (2017). Antiproliferation effect of imatinib mesylate on MCF7, T-47D tumorigenic and MCF 10A nontumorigenic breast cell lines via PDGFR-beta, PDGF-BB, c-kit and SCF genes. Drug Des Devel Ther.

[24] Gojo I, Zhang B, Fenton RG (2002). The cyclin-dependent kinase inhibitor flavopiridol induces apoptosis in multiple myeloma cells through transcriptional repression and down-regulation of Mcl-1. Clin Cancer Res.

[25] Ali MA, Choy H, Habib AA, Saha D (2007). SNS-032 prevents tumor cell-induced angiogenesis by inhibiting vascular endothelial growth factor. Neoplasia.

[26] Kodym E, Kodym R, Reis AE, Habib AA, Story MD, Saha D (2009). The small-molecule CDK inhibitor, SNS-032, enhances cellular radiosensitivity in quiescent and hypoxic non-small cell lung cancer cells. Lung Cancer.

[27] Chen R, Wierda WG, Chubb S, Hawtin RE, Fox JA, Keating MJ (2009). Mechanism of action of SNS-032, a novel cyclin-dependent kinase inhibitor, in chronic lymphocytic leukemia. Blood.

[28] Tong WG, Chen R, Plunkett W, Siegel D, Sinha R, Harvey RD (2010). Phase I and pharmacologic study of SNS-032, a potent and selective Cdk2, 7, and 9 inhibitor, in patients with advanced chronic lymphocytic leukemia and multiple myeloma. J Clin Oncol.

[29] Mariaule G, Belmont P (2014). Cyclin-dependent kinase inhibitors as marketed anticancer drugs: where are we now? A short survey. Molecules.

[30] Stroehlein AJ, Young ND, Korhonen PK, Jabbar A, Hofmann A, Sternberg PW (2015). The *Haemonchus contortus* kinome - a resource for fundamental molecular investigations and drug discovery. Parasit Vectors.

[31] Kovalenko M, Gazit A, Bohmer A, Rorsman C, Ronnstrand L, Heldin CH (1994). Selective platelet-derived growth factor receptor kinase blockers reverse sis-transformation. Cancer Res.

[32] Gazit A, App H, McMahon G, Chen J, Levitzki A, Bohmer FD (1996). Tyrphostins. 5. Potent inhibitors of platelet-derived growth factor receptor tyrosine kinase: structure-activity relationships in quinoxalines, quinolines, and indole tyrphostins. J Med Chem.

[33] Kovalenko M, Ronnstrand L, Heldin CH, Loubtchenkov M, Gazit A, Levitzki A (1997). Phosphorylation site-specific inhibition of platelet-derived growth factor beta-receptor autophosphorylation by the receptor blocking tyrphostin AG1296. Biochemistry.

[34] Banai S, Wolf Y, Golomb G, Pearle A, Waltenberger J, Fishbein I (1998). PDGF-receptor tyrosine kinase blocker AG1295 selectively attenuates smooth muscle cell growth in vitro and reduces neointimal formation after balloon angioplasty in swine. Circulation.

[35] Levitzki A (2013). Tyrosine kinase inhibitors: views of selectivity, sensitivity, and clinical performance. Annu Rev Pharmacol Toxicol.

[36] Karck M, Meliss R, Hestermann M, Mengel M, Pethig K, Levitzki A (2012). Inhibition of aortic allograft vasculopathy by local delivery of platelet-derived growth factor receptor tyrosine-kinase blocker AG-1295. Transplantation.

[37] Iwamoto H, Nakamuta M, Tada S, Sugimoto R, Enjoji M, Nawata H (2000). Platelet-derived growth factor receptor tyrosine kinase inhibitor AG1295 attenuates rat hepatic stellate cell growth. J Lab Clin Med.

[38] Fisher GM, Tanpure RP, Douchez A, Andrews KT, Poulsen SA (2014). Synthesis and evaluation of antimalarial properties of novel 4-aminoquinoline hybrid compounds. Chem Biol Drug Des.

[39] Edgar RC (2004). MUSCLE: a multiple sequence alignment method with reduced time and space complexity. BMC Bioinformatics.

[40] Yang J, Zhang Y (2015). Protein structure and function prediction using I-TASSER. Curr Protoc Bioinformatics.

