# Supplementary material for: Screening of the ‘Stasis Box’ identifies two kinase inhibitors under pharmaceutical development with activity against Haemonchus contortus
Source: Parasit Vectors. 2017 Jul 5;10:323. doi: 10.1186/s13071-017-2246-x (PMC5499055; doi:10.1186/s13071-017-2246-x)
Supplement: Additional file 1: — Five-second video recordings of exsheathed third-stage larvae (xL3s) of Haemonchus contortus displaying the “coiled” phenotype induced by exposure to AG-1295 (MMV079840; 100 μM). Videos of xL3s exposed to the same concentration of SNS-032 (MMV690767), monepantel, or no-compound were also included for comparison. (PPTX 18505 kb) [file 13071_2017_2246_MOESM1_ESM.pptx]

## Slide 1
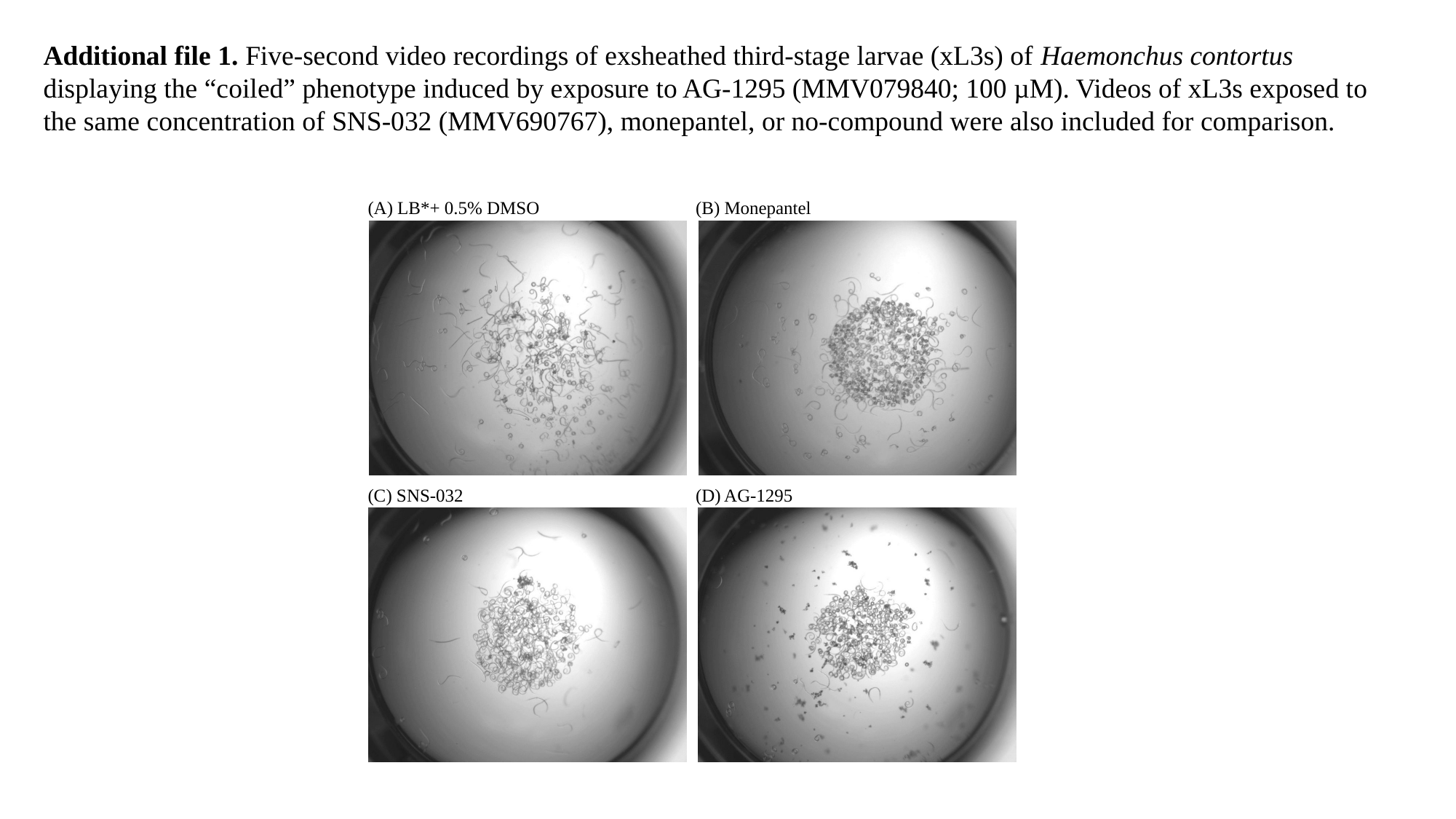

Additional file 1. Five-second video recordings of exsheathed third-stage larvae (xL3s) of Haemonchus contortus displaying the “coiled” phenotype induced by exposure to AG-1295 (MMV079840; 100 µM). Videos of xL3s exposed to the same concentration of SNS-032 (MMV690767), monepantel, or no-compound were also included for comparison.
(B) Monepantel
(A) LB*+ 0.5% DMSO
(D) AG-1295
(C) SNS-032
